# Male New Zealand robins (*Petroica longipes*) cater to their mate’s desire when sharing food in the wild

**DOI:** 10.1038/s41598-017-00879-1

**Published:** 2017-04-18

**Authors:** Rachael C. Shaw, Regan D. MacKinlay, Nicola S. Clayton, Kevin C. Burns

**Affiliations:** 1 School of Biological Sciences, Victoria University of Wellington, PO Box 600, Wellington, 6140 New Zealand; 2 Department of Psychology, University of Cambridge, Cambridge, CB2 3EB UK

## Abstract

In many species that have bi-parental care, food-sharing males provide vital nutritional resources to their mates during reproduction. However, it is currently unknown whether females can signal specific desires to their mates, or if males can cater to female desire in the wild. Here we investigate whether and how wild male North Island robins (*Petroica longipes*) respond to changes in their mates’ desires and nutritional need when sharing food. We demonstrate that wild female robins’ desire for particular foods changes over short time periods; when given the choice between two types of insect larvae, females prefer the type they have not recently eaten. In our experiments, wild male robins preferentially shared the larvae type that their mate was most likely to desire and also increased the quantity of food shared if she had begun incubating. Males catered to their mates’ desire when female behaviour was the only cue available to guide their choices. This is the first evidence that females may behaviourally communicate their specific food desires to their mates, enabling males to cater to fine-scale changes in their mates’ nutritional requirements in the wild. Such a simple behaviour-reading mechanism has the potential to be widespread among other food-sharing species.

## Introduction

Across the animal kingdom, a diverse array of species share food during courtship or copulation^1, 2^. Food sharing with mates (also known as mate provisioning or nuptial gift giving) is typically performed by males and may act as a signal of male quality, and/or may enhance female condition and fecundity^2^. In many bird species, males share food with their mates prior to nesting (courtship feeding) or while females are incubating (incubation feeding). It has been hypothesized that mate provisioning by male birds may provide females with direct benefits, by helping them to meet the nutritional demands of reproduction^3^. Accordingly, food-sharing males may increase feeding rates or the size of food items shared as female nutritional demand increases during incubation^4–8^. In captivity, males have also been shown to attend to the specific type of food the female is most likely to want during courtship feeding^9, 10^. However, it is currently unknown whether females in the wild can signal the type of food that they currently want to their mates, or whether males can, or do, cater to female desire (i.e. transient changes in the subjective value that particular food items hold).

To date, male decisions regarding the type of food to share have only been experimentally examined in captive Eurasian jays (*Garrulus glandarius*)^9, 10^. Researchers investigated whether or not male jays were capable of attributing a mental state, namely desire^11, 12^, to their mates^9^. Jays experience specific satiety^13, 14^; after eating a particular food, their subsequent preference for that food is decreased relative to other foods^9^. Male jays responded to changes in their mates’ desires for particular foods (induced via specific satiety), but only if they had seen what she had eaten immediately prior to sharing^9^. When males did not see what the female had previously eaten, they were unable to choose the appropriate food item to share with her^9^. This result eliminated the possibility that male jays rely on behavioural cues from their mates when deciding what to share with them^9^. Taken together with an additional control experiment, the authors of the jay study argue that their results suggest that jays may be capable of desire-state attribution^9^.

Carefully controlled experiments in captivity can elucidate the mechanisms used by males to cater to their mates’ desires when sharing food^15, 16^. However, it is currently unknown whether and how males in the wild, where there is much more variability in natural food availability, attend to fine-scale changes in their mates’ desire for particular food types. The North Island robin, or toutouwai (*Petroica longipes*), is a small insectivorous passerine endemic to New Zealand. Male robins actively share food with their mates during the breeding season (see video S1 in Supplementary Materials) in a manner that is typical for many food sharing species^17^. Sharing occurs both during courtship and to provision the female while she alone builds the nest and incubates^18^. In addition, wild robins are boldly curious and will readily interact with humans and experimental apparatuses^19, 20^. Thus robins provide an ideal opportunity to evaluate whether males can flexibly adjust both the type and quantity of food shared in response to changing female desire and nutritional need in the wild.

**Figure 1:**
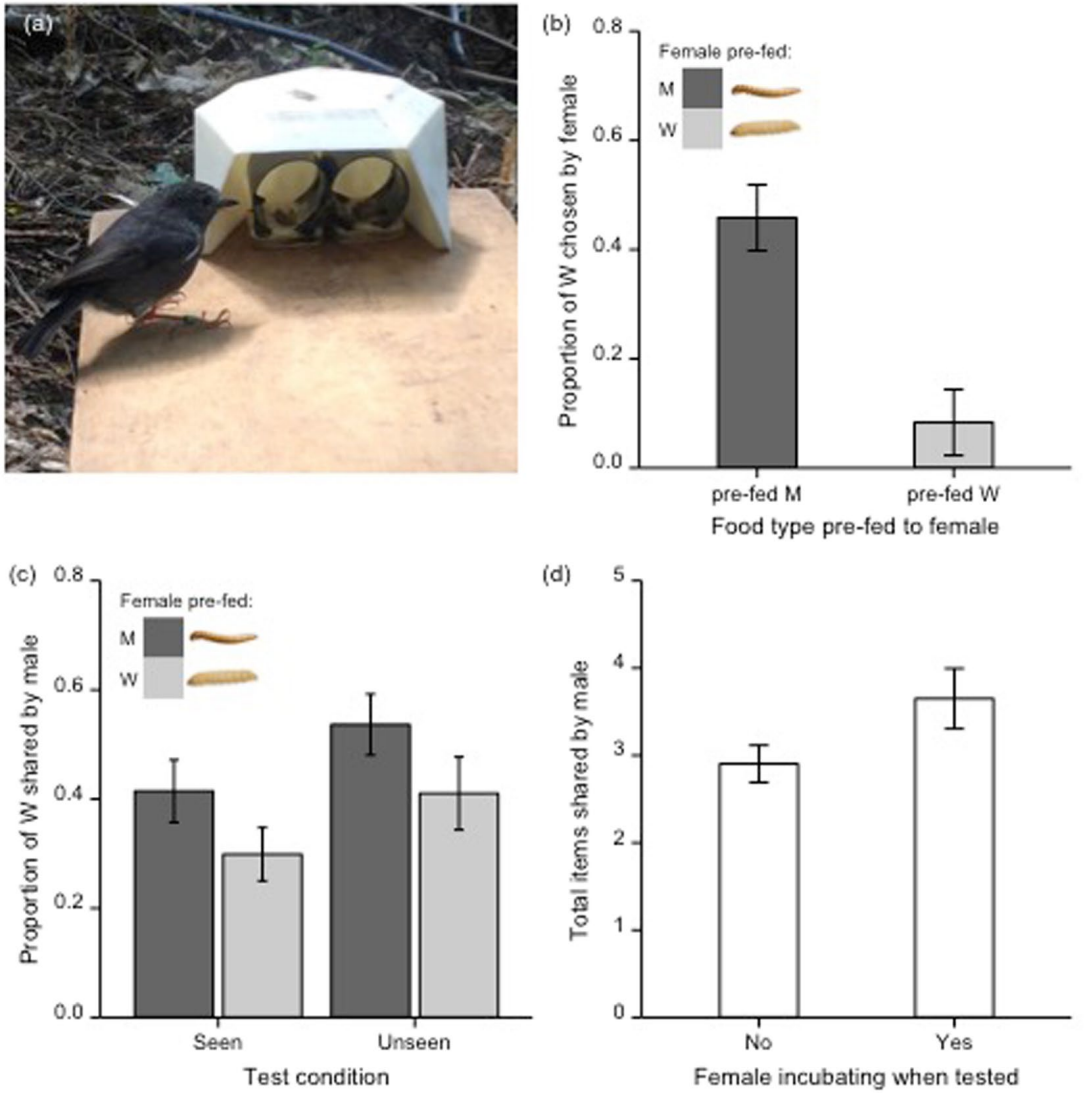
(**a**) The specific satiety test apparatus ensured that the male did not see the female’s choices if he appeared during a trial; the food was presented inside black opaque tubes that were covered by a white barrier. (**b**) In the specific satiety experiment the females chose a larger proportion of W (wax moth larvae) in their first three choices after they had received M (mealworms; dark grey bar) during pre-feeding, compared to after they had received W (light grey bar) during pre-feeding. (**c**) In the food-sharing experiment the males shared a larger proportion of W after the female had been pre-fed M (dark grey bar), compared to after she had been pre-fed W (light grey bar). This pattern of sharing was similar across both the seen and the unseen conditions. (**d**) During the food-sharing experiment the males shared more items if females were incubating than if they were not. In all graphs error bars show the SE of the mean, adjusted for repeated measures where appropriate.

The aim of this study was to investigate whether and how wild male robins cater to changes in their mates’ desire when sharing food. To achieve this we tested 16 pairs of wild robins. We first established whether female robins experience specific satiety to two types of insect larvae (the ‘specific satiety experiment’). We gave the female either ~0.5 g of wax-moth larvae (*Galleria mellonella*; pre-fed W) or ~0.5 g of mealworms (*Tenebrio molitor*; pre-fed M), before offering females the choice between 3 W and 3 M (Fig. 1a). If females experience specific satiety, we predicted that they should choose fewer W in their first three choices after being pre-fed W, compared to when they had been pre-fed M. Subsequently, we tested whether males could respond to changes in female desire using an experimental paradigm that has previously only been used in captivity^9^. In the ‘food-sharing experiment’ we began each trial by pre-feeding the female ~0.5 g of food (either M or W). Immediately after pre-feeding the female, we gave the male six choices between 1 W and 1 M. We allowed the male to take one item and waited for him to share it with the female, eat it, or cache it, before offering him the next choice between 1 W and 1 M. If males respond to changes in their mate’s specific satiety, we predicted that they should share a smaller proportion of W when females had been pre-fed W, compared to when females had been pre-fed M. To examine the type of information that the male robin uses to cater to female desire we ran two conditions in the food-sharing experiment. In the ‘seen’ condition the male could observe the female eating during pre-feeding. By contrast, in the ‘unseen’ condition, the male did not observe the female during pre-feeding. To achieve this we distracted the male in a location that was out of view of the female (see methods for details). We gave each male four trials in the food-sharing experiment (seen female pre-fed W; seen female pre-fed M; unseen female pre-fed W; unseen female pre-fed M). If males need to see the female eating to know what to share with her, we predicted that the effect of pre-feeding should only be evident in the seen condition, thus there should be a significant interaction between the food-type pre-fed to the female and the condition if this is the case^9^. Finally, the timing of our experiments at the onset of breeding also allowed us to examine whether males adjusted the overall quantity of food delivered to the female in response to her incubation status (the number of days prior to, or post incubation onset).

## Results

The female robins experienced specific satiety; eating a particular food decreased their subsequent preference for that food. Compared to when they were pre-fed M, when females were pre-fed W they chose fewer W in their first three choices (Table 1; Fig. 1b). In the food-sharing experiment, males responded to changes in the female’s desire for W and M (induced via specific satiety), regardless of whether or not he had seen the female eating during pre-feeding (as indicated by the lack of a significant interaction between condition and the type of food pre-fed to the female^9^; Table 2a). Compared to when the female was pre-fed M, when the female was pre-fed W the male shared a smaller proportion of W with her (Table 2a; Fig. 1c). Males also responded to the incubation status of the female; if the female was incubating the male shared more food during a trial (Table 2b; Fig. 1d).

**Table 1:** Generalized linear mixed model (GLMM) results for the factors affecting the number of W chosen in the first three choices made by females in the specific satiety experiment.

	Wald *Z*	*P*	Estimate	SE
Food pre-fed to female	−3.586	0.000335	−2.3502	0.6553
Trial order	0.749	0.454		

Data were fitted with a binomial error structure with a logit-link function. We specified two fixed factors: the food pre-fed to female (W or M, with M set as the reference value) and trial order (Trial 1 or 2). We included female ID as a random factor in the model to control for repeated measures (estimated variance component for female ID = 0.494 ± 0.703 SD).

**Table 2:** GLMM results for the factors affecting (a) the number of W shared by the male out of the total items shared during a trial and (b) the total number of items shared by the male. Coefficient estimates and their associated SE are shown for significant terms.

		Wald *Z*	*P*	Estimate	SE
a	Food pre-fed to female	−2.068	0.0387	−0.9563	0.4625
	Days pre/post incubation	−1.682	0.0927		
	Condition	0.674	0.501		
	Pre-fed*Condition	0.579	0.563		
b	Days pre/post incubation	3.243	0.00118	0.0282	0.0087
	Pre-fed	0.821	0.417		
	Pre-fed*Condition	−0.330	0.742		
	Condition	0.265	0.791		

Data were fitted with (a) binomial error structure with a logit-link and (b) Poisson error structure with a logarithmic link function. Both models included the following fixed factors: food pre-fed to female (W or M, with M set as the reference value), condition (‘seen’ set as reference), the interaction of these two factors, as well as the number of days the trial took place pre/post female incubation onset. We fitted male ID as a random factor in each model to control for repeated measures (estimated variance component ± SD for male ID in (a) = 0.319 ± 0.565 and in (b) = 0 ± 0 SD).

There was no effect of pre-feeding on the male’s own specific satiety; males consumed a similar proportion of W across trials, regardless of whether the female had been pre-fed W or pre-fed M (Wald *Z* = −1.186, *P* = 0.236; see Table S1 in ESM for full GLMM results). Prior to all sharing events (203 in total during the experiment), the male carried food away from the test location in the direction of the female and either perched and sang while holding the food item until she approached him, or approached the female directly to initiate sharing. Females always took the food that was offered by the male. When the males chose food items during the test, the female was <4 m away during 151 of 384 choices (39% of all choices) and was <4 m away prior to 105 of 203 sharing events (52% of all sharing events). The male was more likely to share an item with the female when she was <4 m away from him as he chose it, when the female was incubating, or when his choice occurred later in the trial (Table 3).

**Table 3:** GLMM results for the factors affecting the likelihood that a male would share the item with the female after each choice. Coefficient estimates and their associated SE are shown for significant terms.

	Wald *Z*	*P*	Estimate	SE
Female < 4 m from platform	5.104	3.33 × 10^−7^	1.3800	0.270
Days pre/post incubation	4.143	3.43 × 10^−5^	0.0789	0.0190
Choice sequence	2.229	0.0258	0.1549	0.0695
Pre-fed	0.329	0.742		
Condition	−0.009	0.993		

Data were fitted with binomial error structure with a logit-link. The model included the following fixed factors: the distance of the female from platform during a choice (‘1’ when female <4 m away, ‘0’ when female >4 m away; 0 set as reference), the number of days the trial took place pre/post female incubation onset, the sequence of choices within the trial (1–6), the food pre-fed to female and the condition. We fitted male ID as a random factor to control for repeated measures (estimated variance component ± SD for male ID = 0.122 ± 0.349).

## Discussion

Male North Island robins actively initiated sharing events with their partners and flexibly adjusted both the type and quantity of food that they shared to cater to the female’s current desire and incubation status. The males’ sharing choices in the food-sharing experiment followed a similar pattern to the females’ choices in the specific satiety experiment. However, it is possible that males had a preference for eating M (as they had more prior experience with this food^19, 20^) and that this affected their ability to cater to their mate’s current desire, as males shared a larger proportion of W than a female was likely to have chosen for herself after eating W (this difference is evident in a comparison of the pre-fed W trials in Fig. 1b and c). Interestingly, previous research has shown that when male Eurasian jays’ desires conflict with their partner’s, they are also less proficient at catering to female desire^10^. Male sharing behaviour was consistent between the seen and unseen conditions. In the unseen condition, the male did not see what the female had eaten during pre-feeding and so the only cue available to guide his sharing choices was his mate’s behaviour, as affected by her current desire. Thus, in contrast to male Eurasian jays, rather than inferring the female’s desire from a knowledge of what he had previously seen her eat (which the authors of the jay study suggest could indicate that jays may be capable of desire state attribution^9^), a male robin’s sharing choices were instead likely to have been influenced by his mate’s behaviour during the test. For example, the female’s proximity when the male held a desirable food item might have been one such behavioural cue triggering sharing.

Male robins catered to their mate’s changing desires for W or M, regardless of whether they had seen the female eating during pre-feeding. While this result is consistent with the suggestion that males sharing choices were guided by behavioural cues from their mate, an alternative possibility is that males observed their mate eating in the unseen condition, despite our best efforts to prevent this from happening. If this was indeed the case, then we cannot exclude the possibility that male robins, like Eurasian jays^9^, require a knowledge of what their mate has eaten previously to know what to share with her. However we consider that this is an unlikely explanation for the male robins’ sharing behaviour for two reasons. Firstly, if the male needs to know what his mate has previously eaten to attribute a change in her internal desire-state and share the appropriate item, this is arguably a ‘higher-level’ explanation than our proposed alternative, namely that the female’s behaviour directly influences the male’s decision to share an item with her^21^. Secondly, males never attempted to approach the female or the food during pre-feeding in the unseen condition, despite the fact they typically dominate and displace their mates at food resources throughout the year^22, 23^. This indicates that males could not see the food during pre-feeding in the unseen condition.

Sharing was always initiated by the male carrying food away from the test platform and toward his mate, or a singing post. The female was frequently at a distance greater than 4 m from the test platform as the male chose an item (i.e. she was >4 m away during 61% of all choices), but the male was more likely to share with his mate if she was in close proximity (<4 m away). This suggests that a female was more effective at eliciting male sharing behaviour when clearly visible to her mate, either because she noticeably signalled her desire for a food item held by the male (e.g. via begging displays), or because her presence alone was sufficient to trigger increases in male sharing behaviour. Prior to a sharing bout, females typically adopted a begging posture and emitted vocalizations similar to those made by juvenile robins. However, as females often moved position during each trial, we were unable to consistently measure female begging behaviour and intensity in response to the male’s food choices. As we could not directly quantify female behaviour in the current study, identifying the precise behavioural cues that enable males to cater to the females’ desire remains an exciting avenue for future research. Prior to 48% of sharing events, the female was >4 m away from the male, which could have limited her ability to directly signal her desire to share. It is possible that the male’s sharing choices when his mate was at a distance may have been influenced by the female’s behavioural responses during previous sharing events. For example, if the female rapidly approached a singing post when the male held a preferred food item, or more intensely begged prior to or during a sharing bout, the male may have been more likely to share subsequent food items and to choose items that were preferred by his mate. Under natural conditions a male robin may often need to decide whether or not to share an item without any direct cues from his mate, as the male typically calls his mate from the nest to share a food item with her during incubation feeding^18^.

In addition to attending to the type of food that his mate desired, the male also increased the quantity of food that he shared if his mate was incubating. Female nutritional need is likely to be greater during incubation, as the female is limited in the amount of time that she can spend off the nest foraging^18^. It is possible that female robins convey their increased nutritional demands via more intense begging, although we were unable to directly measure begging intensity in the current study. However, a study of incubation feeding in wild pied flycatchers (*Ficedula hypoleuca*) experimentally increased female energetic requirements by clipping primary flight feathers, which led to increases in both female begging intensity and male incubation feeding rates^7^.

Our results suggest that it is likely that a female robin’s behaviour informs the male’s decisions about both the type and quantity of food that he should share. Food-sharing with mates can increase breeding success in socially monogamous species with bi-parental care, particularly when sharing occurs post-copulation^2, 3^. The male robin’s ability to share food that is likely to match his mate’s current needs and desires provides additional support for the hypothesis that food-sharing provides females with direct fitness benefits via compensating for the nutritional limitations imposed by reproduction^2, 3^. When females can signal their dietary requirements to their mates and males can cater their sharing choices to meet these needs, then the fitness benefits of food-sharing may be optimized. Additionally, a male’s food-sharing performance may enable the female to evaluate the quality of her mate, ultimately influencing pair bond dynamics^24^. To further evaluate these possibilities, future studies of food-sharing in the wild could investigate the potential fitness correlates of male food sharing ability. For example, if the ability to cater to a mate’s desires provides males with direct benefits, then it could be expected that individual males that most closely match their mate’s desires may establish pair bonds more quickly, or may have the lowest rates of extra-pair copulation by their mates. The performance of male North Island robins in our experiments suggests that a female behaviour may guide male food-sharing choices in the wild, although the precise behavioural cues involved are yet to be identified. Nonetheless, this relatively simple behaviour-reading mechanism^21^ may be taxonomically widespread, enabling males in many other food-sharing species to cater to the females’ specific nutritional requirements during reproduction.

## Methods

### Study site and subjects

Between 30^th^ September and 13^th^ November 2015 we tested 16 pairs of individually banded wild adult robins (age range 2–8 years) at Zealandia Wildlife Sanctuary in Wellington, New Zealand. The study site and population has been described in detail elsewhere^20^. The research was approved by the Victoria University of Wellington Animal Ethics Committee and conducted under permit from the Department of Conservation (Authorization number: 38497-FAU).

Tests took place on a pair’s breeding territory between 8:30 am and 2:00 pm, on days with minimal rain and wind. Robins typically maintain pair bonds over several breeding seasons^25^ and 15 of the pairs had been together during the previous breeding season. For all pairs, the male and female had shared adjacent territories during the winter prior to testing. We followed the methods of ref. 26 and checked all pairs every second day (or daily) to determine when the female had begun incubating their first clutch of the season.

### Specific satiety experiment

The specific satiety experiment examined whether eating a particular food led to a subsequent decrease in the female’s preference for that food. We tested females between September 30^th^ and October 20^th^ 2015. Only one of the 16 females tested was incubating during the experiment (she was tested in the first two days after incubation began), all other females were not yet incubating. We only tested females when the male was not in view. To further ensure that the male could not observe the female’s choices, we always placed food in the centre of an opaque tube, under a small opaque barrier (tubes were 5 cm in length, 4 cm in diameter and open at both ends, see Fig. 1a). Robins were habituated to the presence of the experimenters^19, 20^ and were already familiar with this apparatus^19^. The female could reach into the open end of a tube to retrieve food. The experimenter (RS) sat at least 1 m away from the testing location and filmed trials using an iPad Air®.

We began each trial with ‘pre-feeding’, where we gave the female ~0.5 g of food. We tested females in two conditions: in one females were pre-fed 3 wax moth larvae (pre-fed W) and in the other females were pre-fed 5 medium sized mealworms (pre-fed M). Immediately after pre-feeding we tested female specific satiety by giving the female two tubes placed side by side: one contained 3 W and the other 3 large M (Fig. 1a). The side that food was presented on was randomized between females, but remained consistent across trials for an individual female. Trial order (pre-fed M and pre-fed W) was counter-balanced between females. We tested females over two sequential days (one trial per day).

As individual M were typically smaller than W it was not always feasible to match the exact quantity and size of W and M offered (due to limited numbers of sufficiently large M). Thus during pre-feeding we standardized the weight (as opposed to number of items) of food pre-fed to the female across treatments, to ensure that the female’s overall general satiation was consistent between the two pre-feeding treatments. However, as both W and M were presented simultaneously during the test phase, in both the specific satiety experiment and the food sharing experiment described below we instead ensured that the total number and item size of each food type were as closely matched as possible. All robins were familiar with M^19, 20^, but had never encountered W. We therefore gave each robin 1 W at least one day prior to their first experiment, to ensure that both males and females were familiar with W and would consume it.

To assess whether females experienced specific satiety to W and M, we investigated the effect of pre-feeding on the number of W that the females chose when we gave them the choice between 3 W and 3 M. We limited this analysis to the first three choices made by females, as trials where females initially chose the same food type three times in a row left only one type of food remaining for the final three choices. We used a Generalized Linear Mixed Model (GLMM) with a binomial error structure and logit link to test whether pre-feeding affected the number of W taken in the first three choices. We included pre-fed food type (W or M) and trial order (1 or 2) as fixed factors and female ID as a random factor to control for repeated measures. For all analyses reported in this study alpha was 0.05 and we analysed data in R (version 3.1.1), using the package lme4^27^ to run GLMMs. All raw data is provided in the ESM Tables S2, S3 and S4.

### Food-sharing experiment

We tested male food-sharing behaviour between October 5^th^ and November 13^th^ 2015, after their mate had been tested in the specific satiety experiment. We began each trial by pre-feeding the female ~0.5 g of food (either 5 medium sized M or 3 W). In the ‘seen’ condition the male observed the female eating. Male robins always dominate food resources and during the breeding season males will frequently displace their mate from a food source and then choose to share the commandeered food with her^22, 23^. To ensure that the male could not displace the female and steal the food, we fed the female when the male was in her direct line of sight, but at least 2 m away. This distance ensured that if the male tried to approach and displace the female, she would have sufficient time to eat all food items before he reached her. In the ‘unseen’ condition, the male was at least 2 m away, with his view of the female blocked by an experimenter (RM) who played the role of a distractor for the male. If necessary, distraction was achieved by throwing a small stick a short distance in a downslope direction, away from the visual line of sight the female (the male would typically follow the stick and closely investigate the area where it had been thrown). Males never attempted to approach the females or food during the ‘unseen’ condition, indicating that they did not observe the female eating. The distractor was also in close proximity to the male in the ‘seen’ condition, but was not blocking his view. Males frequently approached the female and food during pre-feeding in the ‘seen’ condition. The distractor filmed all trials on an iPad Air®.

Immediately after pre-feeding the female, we gave the male six choices between 1 W and 1 M. The experimenter (RS) sat facing the male and placed two transparent tubes at arm’s-length away from herself on the ground, spaced ~10 cm apart. Each tube had an opening facing toward the male and contained either 1 M or 1 W. After the male took an item from one tube, the experimenter removed both tubes and waited for the male to either eat, share, or cache his chosen item. We repeated these steps to give the male a total of six choices between 1 M and 1 W.

In each trial we pseudo-randomized the location of the W and M between the left and right tubes, to ensure that each food type was presented on each side three times during a trial. We gave males four trials in the food-sharing experiment (seen female pre-fed W; seen female pre-fed M; unseen female pre-fed W; unseen female pre-fed M). The order of the seen and unseen conditions was counterbalanced between males and the order of female pre-feeding (W or M) was counter-balanced within each condition. We tested a pair once per day and ran the trials for a pair on sequential days (the weather and presence of both pair members permitting) until the experiment was complete.

If males can respond to female specific satiety we predicted that they should share a smaller proportion of W when females had been pre-fed W, compared to when females had been pre-fed M. Thus there should be an effect of pre-feeding on the proportion of W shared by males. In addition, if a male needs to see the female eating to know what to share with her, the effect of pre-feeding should only be evident in the seen condition, thus there should be a significant interaction between the food-type pre-fed to the female and the condition if this is the case^9^. We tested these predictions using a GLMM with a binomial error structure and a logit link. The response variable in the model was the number of W shared out of all items shared by the male in a trial. We included the following fixed factors: the food pre-fed to the female (W vs. M), the condition (seen vs. unseen), the interaction between pre-feeding and condition and the number of days that the test took place prior to or post incubation onset for the female. Male ID was included as a random factor. We also investigated the effect of these same factors on the total number of items shared by the male in a trial, using a GLMM with a Poisson error structure and a logarithmic link.

To test whether the male’s own satiety may have been affected by his mate’s behaviour during the test, we ran a GLMM to investigate whether pre-feeding, condition, or the interaction of these terms influenced the males own eating behaviour (the number of W consumed out of all items eaten by the male) during a trial (binomial error structure, Male ID included as a random factor). Finally, we used the trial videos to estimate the distance of the female from the platform as the male chose an item (coded as <4 m or >4 m) and record whether the male left the test site to approach the female, or she approached him at the test site before a sharing bout. We used a GLMM to investigate the factors influencing the likelihood that the male would share an item with the female (binomial error structure, Male ID included as a random factor). We included the following fixed factors in the model: the proximity of the female as the male chose an item (<4 m or >4 m from the male), the food pre-fed to the female (W vs. M), the condition (seen vs. unseen), the number of days that the test took place prior to or post incubation onset for the female and the sequence of choices within the trial (1–6).

## Electronic supplementary material

Video S1

ESM containing Table S1

Table S2

Table S3

Table S4
