# Supplementary material for: Male New Zealand robins (Petroica longipes) cater to their mate’s desire when sharing food in the wild
Source: Sci Rep. 2017 Apr 18;7:896. doi: 10.1038/s41598-017-00879-1 (PMC5429848; doi:10.1038/s41598-017-00879-1)
Supplement: Supplementary file 2 — ESM containing Table S1 [file 41598_2017_879_MOESM2_ESM.pdf]

Male New Zealand robins (*Petroica longipes*) cater to their mate's desire when sharing food in the wild

Rachael C. Shaw, Regan D. MacKinlay, Nicola S. Clayton and Kevin C. Burns

Video S1. An example of a male North Island robin sharing food with his mate.

Table S1. GLMM analysis of the factors affecting the number of W eaten out of all items eaten by the male during the food-sharing experiment.

	Wald *Z*	*P*
Condition	-1.730	0.084
food pre-fed to female	-1.186	0.236
food pre-fed to female *condition	1.000	0.317

Data were fitted using a binomial distribution with a logarithmic link function. We specified the food pre-fed to female (W or M, with M set as the reference value), condition (seen vs unseen, with seen set as reference) and the interaction of these terms as fixed factors. We included male ID as a random factor in the model to control for repeated measures (estimated variance component for male ID = 0.212 ± 0.460 SD).
